# Aging-Related Endothelial Progenitor Cell Dysfunction and Its Association with IL-17 and IL-23 in HFmrEF Patients

**DOI:** 10.1155/2022/2281870

**Published:** 2022-06-26

**Authors:** Lijin Zeng, Cong Zhang, Guoyi Cai, Bin Zhang, Zixia Huang, Mingyue Wu, Yuanting Zhu, Liang Luo, Hao He, Zhen Yang

**Affiliations:** ^1^Division of Emergency Medicine, The First Affiliated Hospital, Sun Yat-sen University, Guangzhou, China; ^2^Department of Cardiology, The First Affiliated Hospital, Sun Yat-sen University, Guangzhou, China; ^3^NHC Key Laboratory on Assisted Circulation (Sun Yat-sen University), Guangzhou, China; ^4^Department of Cardiovascular Disease, Jiangmen Central Hospital, Affiliated Jiangmen Hospital of Sun Yat-sen University, Jiangmen, China; ^5^Clinical Experimental Center, Jiangmen Central Hospital, Affiliated Jiangmen Hospital of Sun Yat-sen University, Jiangmen, China; ^6^Department of Anesthesiology, The Second Affiliated Hospital, Nanhua University, Hengyang, China; ^7^Department of Critical Care Medicine, Seventh Affiliated Hospital, Sun Yat-sen University, Shenzhen, China; ^8^Department of Cardiology, The Fifth Affiliated Hospital of Zunyi Medical University, Zhuhai, China

## Abstract

**Background:**

Aging is an independent risk factor for heart failure (HF), and endothelial progenitor cell (EPC) function decreases with aging. Here, we further investigated whether age has a detrimental effect on circulating EPC function in HF with mildly reduced ejection fraction (HFmrEF) and its relationship with systemic inflammation.

**Methods:**

58 HFmrEF patients were recruited. The adhesive, migrative, and proliferative activities of circulating EPCs, MAGGIC scores, and plasma interleukin (IL)-17 and IL-23 levels of these patients were assessed.

**Results:**

Older patients with HFmrEF had higher MAGGIC scores and lower circulating EPC adhesion, migration, and proliferation than younger patients. The similar tendency was observed in plasma IL-17 and IL-23 levels. The EPC functions were negatively associated with MAGGIC scores and plasma IL-17 or IL-23 levels.

**Conclusions:**

In patients with HFmrEF, aging leads to attenuated circulating EPC function, which is correlated with disease severity and systemic inflammation. The present investigation provides some novel insights into the mechanism and intervention targets of HFmrEF.

## 1. Introduction

Heart failure (HF) is the leading cause of cardiovascular mortality and places great stress on public health systems globally [1, 2]. Its incidence and mortality increase significantly with age [3]. The 2021 HF guidelines define HF with mildly reduced ejection fraction (HFmrEF) as having an ejection fraction of 41–49% [4], while it was defined as the EF of 40-49% in the 2016 European Society of Cardiology (ESC) Guidelines [5]. HFmrEF accounts for 10–24.2% of HF patients [6–8] and has an all-cause mortality 2–3 times higher in the elderly [9]. The Meta-analysis Global Group in Chronic Heart Failure (MAGGIC) score includes 13 independent predictors of mortality and predicts the 1- and 3-year all-cause mortality of HF [10, 11]. Although many studies have examined the characteristics of HFmrEF since it was first named in the 2016 ESC guidelines [5], the effect of aging on MAGGIC score in HFmrEF patients is still unknown.

Endothelial progenitor cells (EPCs) derived from bone marrow help repair injured endothelium by differentiating into endothelial cells [12]. The EPC level is reduced in advanced HF [13] and is an independent predictor of mortality in HF [14, 15]. A recent study reported that the number and migratory capacity of EPCs did not decrease with age in patients with HF with a reduced ejection fraction (HFrEF) [16]. However, the influence of aging on circulating EPCs in HFmrEF is unclear.

Aging is closely associated with inflammation, which is mainly induced by damage-associated molecular patterns (DAMPs) via interactions with cell receptors and the upregulation of proinflammatory cytokines [17, 18]. The severity of HF is positively correlated with proinflammatory cytokine levels [19, 20], which are also independent predictors of cardiovascular events in older patients [21]. IL-17 is an important proinflammatory cytokine that increases with age and is related to the New York Heart Association (NYHA) class of HF [22, 23]. IL-17 is maintained mainly by IL-23, another cytokine that induces Th17 differentiation [24]. Previously, we found that IL-17 was negatively related to EPC function in older non-ST segment elevation myocardial infarction patients [25]. We hypothesized that increasing IL-17 and IL-23 might be the mechanism underlying the alteration in function of circulating EPCs in aging patients with HFmrEF. Therefore, we assessed the variation in MAGGIC score function of circulating EPCs, and plasma IL-23 and IL-17 levels in older and younger HFmrEF patients, and analysed the correlations between them.

## 2. Methods

### 2.1. Study Population

Patients diagnosed with HFmrEF were divided into older (≥65 years) and younger (<65 years) groups. The HFmrEF inclusion criterion was a clinician-judged HF with a left ventricular ejection fraction (LVEF) of 41–49% [4]. All patients were older than 18 years and provided venous blood to assess the quality of circulating EPCs and plasma IL-17 and IL-23 levels.  Table 1 lists the basic clinic characteristics.

### 2.2. MAGGIC Score Calculation

MAGGIC scores which included age, gender, EF, body mass index, systolic blood pressure, time since HF diagnosis, NYHA class, serum creatinine, chronic obstructive pulmonary disease, diabetes, current smoking, and current therapy with beta-blocker, angiotensin-receptor blockers, and ACE-inhibitor were calculated as reported previously [11].

### 2.3. EPC Function

The adhesion, migration, and proliferation of circulating EPCs were assessed as reported previously [26–29]. Briefly, EPC adhesion was determined by fibronectin-coated culture plates as reported previously [26]. EPC migration was evaluated by modified Boyden chamber containing EBM-2 and supplemented with vascular endothelial growth factor (VEGF) as reported previously [27, 28]. EPC proliferation was analysed by 3-(4,5-dimethylthiazol-2-yl)-2,5-diphenyltetrazolium bromide, (MTT) assay [27–29].

### 2.4. Evaluating IL-17 and IL-23

Enzyme-linked immunosorbent assay (ELISA) was performed using Human IL-17 ELISA Kit (R&D Systems, Inc., Minneapolis, MN, USA) and Human IL-23 ELISA Kit (R&D Systems, Inc., Minneapolis, MN, USA) to measure the plasma IL-17 and IL-23 levels according to the manufacturer's instructions.

### 2.5. Statistical Analysis

SPSS ver. 23.0 (SPSS, Chicago, IL, USA) was used to analyse the statistics. The differences between the groups with nonnormally distributed data were compared with the Mann–Whitney *U*-test, while the Student's *t*-test was used for normally distributed data. Correlations of normally distributed data were analysed with Pearson's test, and nonnormally distributed data were analysed with Spearman's test. Statistical significance was defined as *P* < 0.05.

## 3. Results

### 3.1. Baseline Characteristics

The study enrolled 25 younger (<65 years) and 33 older (≥65 years) HFmrEF patients.  Table 1 lists the baseline characteristics and laboratory results of all patients. Characteristics including sex, heart rate, blood pressure, BMI, and laboratory findings including creatinine, plasma glucose, HDL, LDL, triglycerides, cholesterol, NT pro-BNP, septal E', left ventricular end-diastolic diameter, tricuspid annulus systolic plane excursion, and LVEF were comparable between the younger and the older HFmrEF group (all *P* > 0.05). MAGGIC score was higher in older group than that in younger group (*P* < 0.05).

### 3.2. Differences in EPC Function between Younger and Older HFmrEF Patients

**Figure 1:**
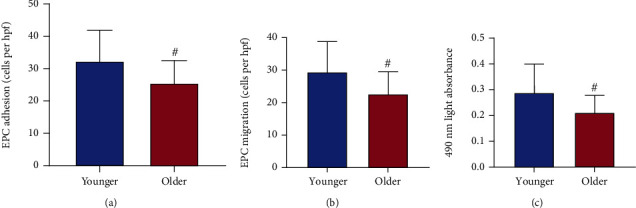
EPC function of younger and older patients. The adhesion (a), migration (b), and proliferation (c) of circulating EPCs decreased in older HFmrEF patients. Data were given as mean ± SD and compared with the Student's *t*-test. ^#^*P* < 0.05*vs*. younger group.

As the adhesion of circulating EPCs was evaluated by fibronectin-coated culture plates, the EPC adhesion was reduced in older HFmrEF patients when compared with younger HFmrEF patients (Figure 1(a), *P* < 0.05). As the migration of circulating EPCs was tested by modified Boyden chamber, the EPC migration also decreased in older HFmrEF patients in comparison with younger HFmrEF patients (Figure 1(b), *P* < 0.05). Similarly, as the proliferation of circulating EPCs was detected by MTT assay, the EPC proliferation was lessened in older HFmrEF patients (Figure 1(c), *P* < 0.05).

### 3.3. EPC Function Impaired More in the High MAGGIC Score Group

**Figure 2:**
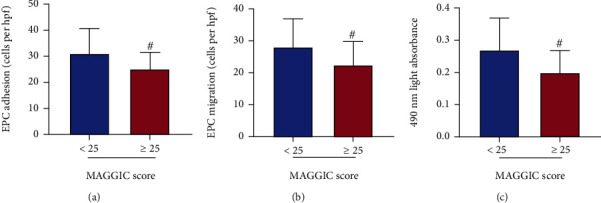
Comparison of EPC function between the high MAGGIC score group and low-score group. The high MAGGIC score group had lower circulating EPC adhesion (a), migration (b), and proliferation (c) than the low-score group. Data were given as mean ± SD and compared with the Student's *t*-test. ^#^*P* < 0.05*vs*. score < 25 group.

The patients were divided into two groups according to whether the MAGGIC score was ≥25 or not, and the circulating EPC function was compared between the two groups. As Figure 2 shows, the adhesive function of circulating EPCs in the high-score group was significantly lower than that in the low-score group (*P* < 0.05). Meanwhile, the migration and proliferation of circulating EPCs in the high-score group were inferior to those in the low-score group (all *P* < 0.05).

### 3.4. Relationship between MAGGIC Score and EPC Function

**Figure 3:**
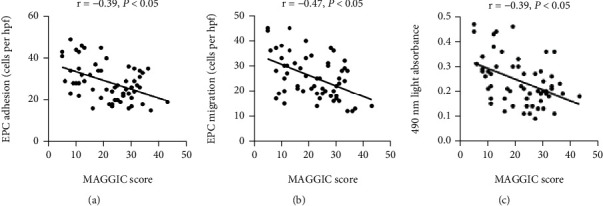
Relationship between MAGGIC score and EPC function. The MAGGIC score was negatively correlated with the circulating EPC adhesion (a), migration (b), and proliferation (c). Correlations of normally distributed data were analysed with Pearson's test, and nonnormally distributed data were analysed with Spearman's *t*est.

As Figure 3 exhibits, the circulating EPC adhesion negatively correlated with the MAGGIC score in the HFmrEF patients (*P* < 0.05). The migration and proliferation of circulating EPCs were also negatively connected with MAGGIC score in the HFmrEF patients (all *P* < 0.05).

### 3.5. Plasma IL-17 and IL-23 Levels in Younger and Older Patients and the MAGGIC Score

**Figure 4:**
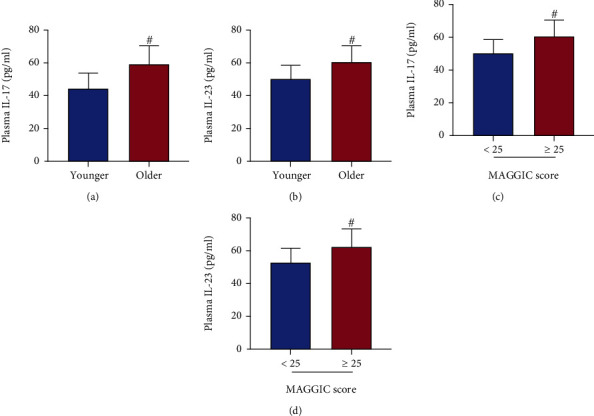
Comparisons of IL-17 and IL-23 levels. IL-17(a) and IL-23 (b) were higher in older patients than in younger ones. Similarly, patients with MAGGIC score ≥ 25 had higher IL17 (c) and IL-23 (d) levels than those in the <25 score group. Data were given as mean ± SD and compared with the Student's *t*-test. ^#^*P* < 0.05*vs*. younger group or < 25 score group.

The plasma IL-17 level was higher in older HFmrEF patients than that in the younger HFmrEF patients (Figure 4(a), *P* < 0.05). Likewise, the plasma IL-23 in the older HFmrEF patients exceeded more than that in the younger HFmrEF patients (Figure 4(b), *P* < 0.05). Moreover, the plasma IL-17 and IL-23 levels were also elevated in the high MAGGIC score group when compared with the low MAGGIC score group (Figures 4(c) and 4(d), all *P* < 0.05).

### 3.6. The Correlation between Inflammatory Cytokines and EPC Function or MAGGIC Score

**Figure 5:**
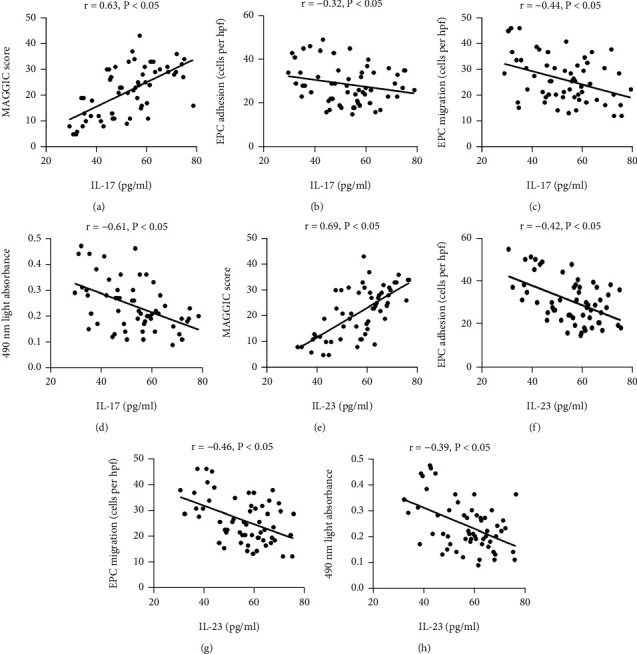
Relationships between proinflammatory cytokines and MAGGIC score or EPC function. MAGGIC score was positively correlated with IL-17 (a) and IL-23 (E). EPC adhesion, migration, and proliferation were negatively related to IL-17 (b)–(d) and IL-23 (f)–(h). Correlations of normally distributed data were analysed with Pearson's test, and nonnormally distributed data were analysed with Spearman's test.

The MAGGIC score was positively related to plasma IL-17 or IL-23 level in the HFmrEF patients (Figures 5(a) and 5(d), all *P* < 0.05). Conversely, the adhesion of circulating EPCs negatively correlated with plasma IL-17 or IL-23 level in the HFmrEF patients (Figures 5(b) and 5(f), all *P* < 0.05). The circulating EPC migrative function was also negatively associated with plasma IL-17 or IL-23 level in the HFmrEF patients (Figures 5(c) and 5(g), all *P* < 0.05). In line with migration and adhesion, plasma IL-17 or IL-23 level had a negative relationship with the circulating EPC proliferation (Figures 5(d) and 5(h), all *P* < 0.05).

### 3.7. The Correlation Between Age and EPC Function, MAGGIC Score, or Inflammatory Cytokines

**Figure 6:**
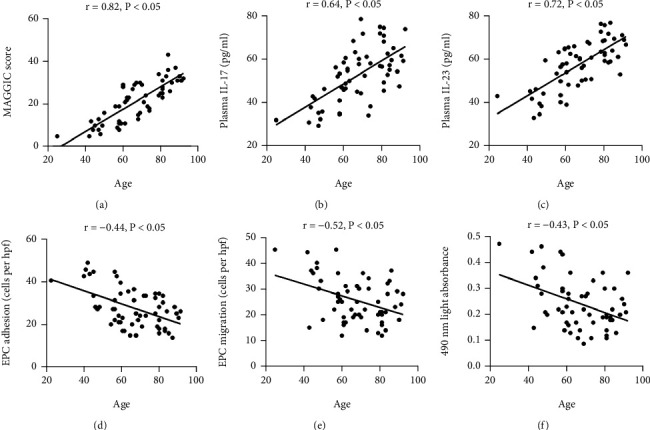
Relationships between age and the MAGGIC score, inflammatory cytokines, or EPC function. Positive relationships were observed between age and the MAGGIC score (a), IL-17 (b), and IL-23 (c), but negative correlations were found between age and EPC adhesion (d), migration (e), and proliferation (f). Correlations of normally distributed data were analysed with Pearson's test, and nonnormally distributed data were analysed with Spearman's test.

Positive relationships were observed between age and MAGGIC score in the HFmrEF patients (Figure 6(a), *P* < 0.05), indicating the effect of aging on disease severity in HFmrEF. Negative correlations between age and EPC adhesive, migrative, and proliferative function were also discovered in the HFmrEF patients (Figures 6(d)–6(f), all *P* < 0.05). Besides that, age was inversely related to plasma IL-17 or IL-23 levels in the HFmrEF patients (Figures 6(b) and 6(c), all *P* < 0.05).

## 4. Discussion

This study revealed an age-related increase in disease severity and reduced circulating EPC function in HFmrEF patients. Similarly, plasma IL-17 and IL-23 levels increased more in older HFmrEF patients than in younger patients and were closely related to disease severity and circulating EPC function. This is the first report of age differences in MAGGIC scores and circulating EPC function in HFmrEF, which is at least partly correlated with the increment in proinflammatory cytokines.

Although aging is an independent predictor of HF prognosis [3], no previous studies have examined how aging affects the severity of HFmrEF. Here, we found that the MAGGIC score, an effective prognostic assessment method for HF, was higher in older HFmrEF patients than in younger ones, implying that aging contributes to the poor prognosis of HFmrEF. It is essential to determine the underlying mechanism.

Previous studies have reported that aging impairs EPC function, which is critical for endothelial repair [30, 31], and that sustained reduction of EPC level contributes to HF via its effect on structural abnormalities [32]. Most research has focused on the relationship between the numbers of EPCs and HF, whereas only a few studies have provided data on HF-related EPC function, and the limited data suggested that EPC activity is impaired in HF [33, 34]. Interestingly, we found that aging aggravated circulating EPC dysfunction in HFmrEF patients, which differed from the findings of Sandri et al., who reported that while EPC function tended downward with age, the difference was not significant [16]. This discrepancy may be mainly due to differences between study populations: Sandri et al. assessed HFrEF patients, and we assessed HFmrEF patients. Our investigation further found that EPC adhesion and proliferation were also mitigated in HFmrEF patients, implying the panorama of age-related qualitative alteration of endothelial repair potential in HFmrEF. In addition, we also found that EPC function was negatively related to the MAGGIC score, indicating that aging worsened the prognosis of HFmrEF partly through impaired EPC function, while increased EPC activity may contribute to improvement in the prognosis of aging HFmrEF patients.

Proinflammatory cytokines increase with age [22, 25, 35], and IL-17 level is associated with the severity and prognosis of HF [23]. Previous studies have implicated the IL-23/IL-17 axis in the development of myocarditis and cardiac remodelling after myocardial infarction [36–38]. Here, we compared plasma IL-17 and IL-23 levels in HFmrEF patients and found that the older HFmrEF group had higher levels than the younger group. Additionally, IL-17 and IL-23 were positively associated with the MAGGIC score, consistent with the results of a previous study reporting a strong correlation between HF and proinflammatory cytokines [23]. Proinflammatory mediators can impair EPC activity partly by regulating NF-*κ*B [39, 40]. Previously, we showed that IL-6 and IL-17 were negatively related to EPC function in non-ST segment elevation myocardial infarction patients [25]. In line with previous reports, our study demonstrated that IL-17 and IL-23 levels were inversely related to EPC function in HFmrEF patients, indicating that the aging-related increment of IL-17 and IL-23 might partly be the mechanism underlying circulating EPC dysfunction in HFmrEF. IL-17/IL-23 axis may be a potential intervention target for the treatment of older patients with HFmrEF.

There are clinical implications in the present study. First, our data indicated that EPC function was more seriously impaired in older HFmrEF patients and was related to disease severity, implying that EPC function may act as a surrogate biomarker for assessing the prognosis of HFmrEF. Recovering EPC function may be a latent therapeutic strategy for older HFmrEF patients. Second, the weakened EPC function may be partly due to the excessive activation of IL-17/IL-23 axis in HFmrEF. Thus, anti-inflammation intervention may be beneficial for the treatment of HFmrEF patients.

Our study had some limitations. First, we did not uncover the exact mechanism underlying the effect of IL-17 or IL-23 on EPC function. Further research is needed to address it. Second, the investigation did not pursue the MACE (Major Adverse Cardiovascular Events) in older HFmrEF patients. Previous research has verified the role of MAGGIC score in predicting the mortality of HF [10, 11]. As the relationship between MAGGIC score and EPC function is revealed by our study, it can be inferred that EPC function may be valuable in predicting the prognosis of HFmrEF.

## 5. Conclusions

This study demonstrated that the activity of circulating EPCs was impaired in older HFmrEF patients, which was related to disease severity. The impaired EPC function in elderly patients with HFmrEF might be partly attributable to systemic inflammation. Our findings suggest that improvement in attenuated endothelial repair ability may be helpful for the treatment of aging HFmrEF.

## Figures and Tables

**Table 1:** Clinical and biochemical characteristics of younger and older patients with HFmrEF.

Characteristics	Younger group (*n* = 25)	Older group (*n* = 33)
Age (years)	58.0 (47.0,60.5)	80.0 (70.5,84.0)^**#**^
Male (*n*, %)	23 (92.0%)	25 (75.8%)
Heart rate (time/min)	80.0 (76.0, 102.0)	80.0 (73.5, 93.0)
Systolic BP (mmHg)	128.2 ± 21.0	127.7 ± 20.7
Diastolic BP (mmHg)	78.5 ± 12.2	72.0 ± 14.0
BMI (kg/m^2^)	20.6 (20.3, 23.9)	20.0 (18.7, 23.7)
Plasma glucose (mmol/L)	7.1 (5.8, 8.7)	7.9 (6.1, 9.5)
Cr (mmol/L)	109.0 (78.5, 225.0)	108.0 (75.5, 184.5)
TC (mmol/L)	4.3 ± 1.9	3.9 ± 1.3
TG (mmol/L)	1.2 (1.0, 2.0)	1.2 (0.8, 1.6)
HDL (mmol/L)	0.9 (0.7, 1.1)	1.0 (0.8, 1.4)
LDL (mmol/L)	2.9 ± 1.2	2.4 ± 0.9
NT pro-BNP (pg/mL)	1883.0 (435.3,30168.5)	4288.0 (817.0,23584.0)
Septal E' (cm/s)	5.0 (4.0,6.5)	5.0 (3.5,6.0)
LVEDD (mm)	56.4 ± 5.2	54.6 ± 7.6
TAPSE (mm)	19.3 ± 4.6	19.6 ± 3.9
LVEF (%)	44.0 (42.0, 46.5)	43.0 (42.0, 45.0)
MAGGIC score	11.0 (8.5,20.0)	29.0 (23.5,32.5)^**#**^

Abbreviations: BMI: Body mass index; BP: Blood pressure; Cr: Serum creatinine; TC: Total cholesterol; TG: Triglyceride; HDL: High-density lipoprotein; LDL: Low-density lipoprotein; LVEDD: Left ventricular end-diastolic diameter; TAPSE: Tricuspid annulus systolic plane excursion; LVEF: Left ventricular ejection fraction; MAGGIC: Meta-analysis Global Group in Chronic Heart Failure. Notes: Normally distributed data are given as the mean ± SD and nonnormally distributed data as the median (25%, 75%). The differences between the groups with nonnormally distributed data were compared with the Mann–Whitney *U*-test, while the Student's *t*-test was used for normally distributed data. ^#^*P* < 0.05*vs*. younger group.

## Data Availability

The original data can be obtained from the corresponding author if it is permitted by all authors.
